# Red Blood Cell Invasion by *Plasmodium vivax*: Structural Basis for DBP Engagement of DARC

**DOI:** 10.1371/journal.ppat.1003869

**Published:** 2014-01-09

**Authors:** Joseph D. Batchelor, Brian M. Malpede, Natalie S. Omattage, Gregory T. DeKoster, Katherine A. Henzler-Wildman, Niraj H. Tolia

**Affiliations:** 1 Department of Molecular Microbiology and Microbial Pathogenesis, Washington University School of Medicine, Saint Louis, Missouri, United States of America; 2 Department of Biochemistry and Molecular Biophysics, Washington University School of Medicine, Saint Louis, Missouri, United States of America; Seattle Biomedical Research Institute, United States of America

## Abstract

*Plasmodium* parasites use specialized ligands which bind to red blood cell (RBC) receptors during invasion. Defining the mechanism of receptor recognition is essential for the design of interventions against malaria. Here, we present the structural basis for Duffy antigen (DARC) engagement by *P. vivax* Duffy binding protein (DBP). We used NMR to map the core region of the DARC ectodomain contacted by the receptor binding domain of DBP (DBP-RII) and solved two distinct crystal structures of DBP-RII bound to this core region of DARC. Isothermal titration calorimetry studies show these structures are part of a multi-step binding pathway, and individual point mutations of residues contacting DARC result in a complete loss of RBC binding by DBP-RII. Two DBP-RII molecules sandwich either one or two DARC ectodomains, creating distinct heterotrimeric and heterotetrameric architectures. The DARC N-terminus forms an amphipathic helix upon DBP-RII binding. The studies reveal a receptor binding pocket in DBP and critical contacts in DARC, reveal novel targets for intervention, and suggest that targeting the critical DARC binding sites will lead to potent disruption of RBC engagement as complex assembly is dependent on DARC binding. These results allow for models to examine inter-species infection barriers, *Plasmodium* immune evasion mechanisms, *P. knowlesi* receptor-ligand specificity, and mechanisms of naturally acquired *P. vivax* immunity. The step-wise binding model identifies a possible mechanism by which signaling pathways could be activated during invasion. It is anticipated that the structural basis of DBP host-cell engagement will enable development of rational therapeutics targeting this interaction.

## Author Summary

Malaria parasites, including *Plasmodium vivax*, must actively invade erythrocytes during blood stage growth in humans. *P. vivax* Duffy Binding Protein (DBP) is a critical invasion ligand that recognizes the receptor Duffy antigen/Receptor for chemokines (DARC) during invasion. To identify critical binding contacts during parasite red blood cell invasion and determine the molecular basis of DBP receptor recognition, we identified the minimal region of DARC contacted by DBP and performed structural studies on the minimal binding domain of DBP in complex with the minimal region from DARC. These studies revealed that two DBP molecules bind two DARC molecules. We performed erythrocyte binding assays with binding site mutants and identified essential receptor contacts. The identification of receptor binding sites and molecular interactions critical to the invasion process provides a basis for targeted disruption of erythrocyte invasion mediated by DBP. The structural and functional studies of DBP and DARC presented here may aid in the rational design of vaccines and invasion inhibitory therapeutics.

## Introduction

*Plasmodium vivax* is a widely distributed human parasite, with 40% of the world's population at risk of infection and an estimated 70–130 million cases of *P. vivax* malaria each year [1], [2]. *P. vivax* is prevalent in India, Southeast Asia, and South America [1], but is rare in most of Sub-Saharan Africa [3]. This rarity is the result of a silencing mutation in the Duffy blood group, found at frequencies near fixation in Sub-Saharan Africa [4], that confers resistance to *P. vivax* [5]. This phenotype has arisen independently at least three times, and *P. vivax* in malaria endemic regions has driven selection for the Duffy negative phenotype. This phenotype confers protection against *P. vivax* because during red blood cell (RBC) invasion the *P. vivax* Duffy Binding Protein (DBP) binds the Duffy antigen/receptor for chemokines (DARC) on RBCs [6], [7]. Therefore, RBCs which lack DARC are refractory to *P. vivax* invasion. DARC is an atypical GPCR, thought to serve as a ‘reservoir’ for excess inflammatory chemokines [8].

Repeated cycles of RBC invasion and rupture cause the clinical symptoms of malaria. To invade a RBC, *Plasmodium* merozoites release the contents of specialized apical organelles: the micronemes and rhoptries. DBP is a member of the erythrocyte binding-like (EBL) family of proteins, which localize to micronemes and use Duffy binding-like (DBL) domains to bind specific RBC receptors with high affinity. DBL domains are located in “region II” of EBL proteins [9], [10], and DBP region II (DBP-RII) is required for formation of a tight junction between *Plasmodium* and RBC membranes. DBP is an exceptional *P. vivax* therapeutic target because it is the sole EBL family member in the *P. vivax* genome [11]. This contrasts with *P. falciparum*, which has multiple, redundant EBL family members which mediate RBC invasion by binding different host RBC receptors.

DBP is a leading vaccine candidate against *P. vivax* malaria [12]. Individuals living in endemic regions develop natural immunity to *P. vivax* in an age-dependent manner which strongly correlates with humoral and cellular recognition of DBP-RII [13]–[15]. Antibodies against DBP inhibit *P. vivax* RBC invasion [16], and antibody epitopes in DBP-RII recognized by inhibitory antibodies have been identified [17]. However, due to a high level of polymorphism in DBP-RII and the selection for strain-specific immunity, identifying residues that are essential to the invasion interaction is still a critical step towards defining vaccination targets.

Previous studies have illuminated key determinants of DBP-RII binding to DARC and begun to define essential elements of the binding interaction. DARC exists as two codominant alleles, *Fya* and *Fyb*, with a single polymorphism at residue 42. Fya contains a glycine, and Fyb an aspartate (G/D42) at this position. The Fyb phenotype has been shown to increase binding to DBP-RII [18]. It is also known that recombinant DARC is sulfated at tyrosine residues 30 and 41, and sulfation of tyrosine 41 has been shown to play a role in binding to DBP [19]. Specifically, a sulfated recombinant DARC N-terminus construct inhibits the DBP-RII erythrocyte interaction to a greater extent than an unsulfated construct. Extensive functional studies have also suggested interaction residues for both DBP-RII and DARC [20]–[25]. The crystal structure of DBP-RII has been solved [26], and illuminated a putative sulfotyrosine binding pocket. Biophysical studies have demonstrated that a non-sulfated DARC construct functionally binds and is capable of inducing dimerization of DBP-RII [26], suggesting that regions outside of the sulfotyrosine residues play an important role in the binding interaction. Despite these studies on the DBP-RII interaction with DARC, the full mechanism of binding and complete extent of molecular interactions between these binding partners are not fully understood.

In an effort to define the mechanism of DBP red blood cell binding and to identify specific molecular interactions at the *P. vivax* invasion interface, DBP-RII was crystallized with the DARC ectodomain. Two crystal forms were observed, a heterotrimeric complex in which one DARC molecule binds the DBP-RII dimer, and a heterotetrameric complex containing two DARC molecules bound to the DBP-RII dimer. The crystal structures of these two complexes represent the first structural characterization of a receptor-bound *Plasmodium* EBL ligand and provide insight into the structure of a portion of the DARC ectodomain that mediates this interaction. The structures illuminate DARC contact residues that explain inter-species barriers to *P. vivax* infection. In addition, point mutations in DARC binding site residues within *P. knowlesi* DBP homologs provide a potential model for the molecular basis of receptor specificity within the EBL family. The heterotetrameric complex shows that the DARC molecules are bound in parallel, resulting in a *Plasmodium* proximal face and a RBC proximal face of DBP-RII. The structures confirm the previously identified DBP-RII dimer interface [26] as a putative target of protective immunity, and reveal novel targets for naturally acquired immunity, including the RBC proximal face of DBP-RII and the DARC-binding pockets. Our studies also provide the basis for a framework defining the mechanism of DARC engagement by *P. vivax*. Specifically, the two bound forms of DBP-RII observed in the crystal structures suggest that an initial binding event followed by receptor-induced DBP dimerization leads to a DBP∶DARC heterotrimer that subsequently binds to a second DARC monomer to create the final heterotetrameric assembly. Isothermal titration calorimetry (ITC) experiments performed with recombinant DARC and DBP-RII support the two state, induced dimerization model of erythrocyte engagement.

## Results

### DBP-RII engages the central region of the DARC N-terminal ectodomain

**Figure 1:**
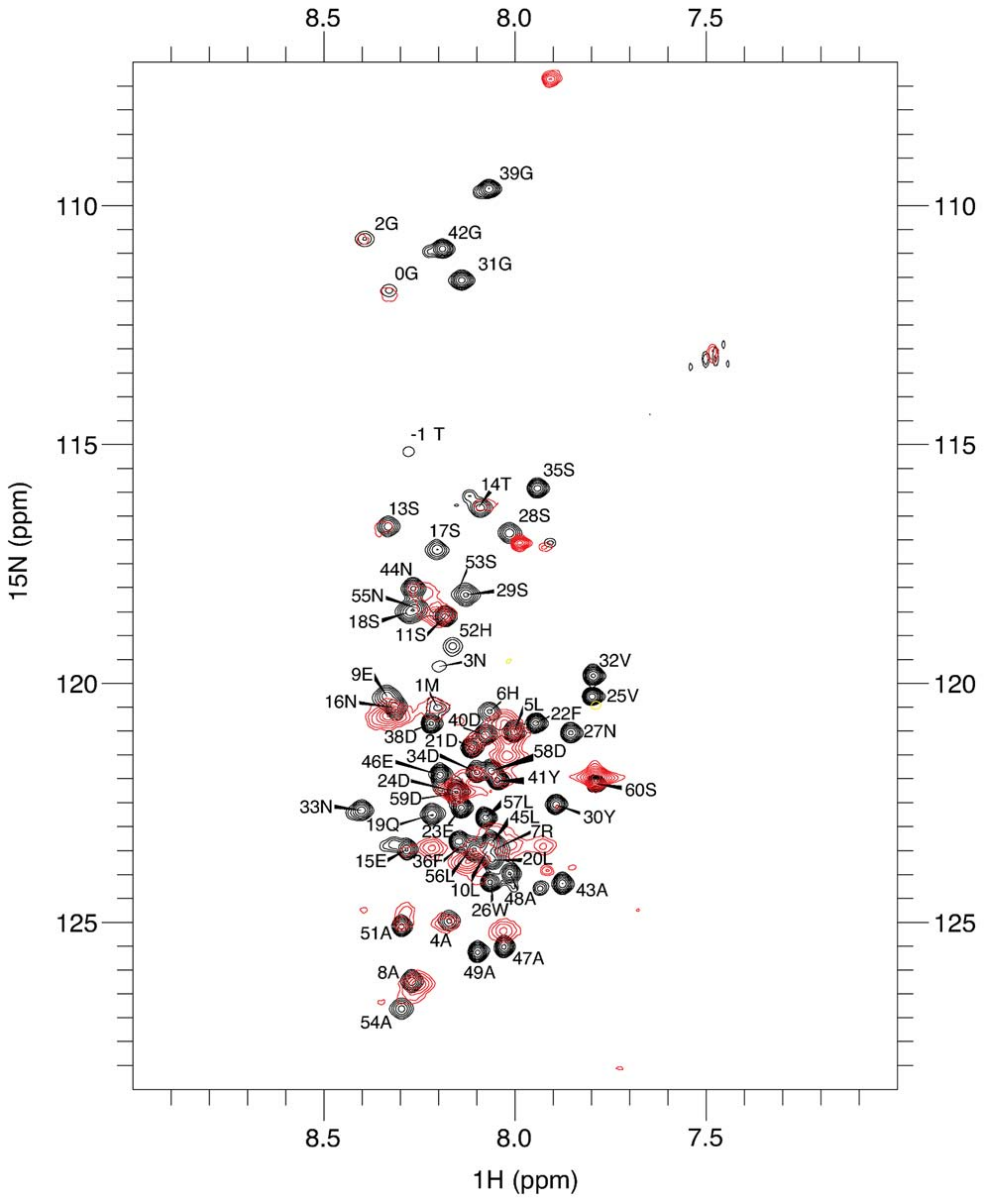
Residues 14–43 of DARC contain the minimal binding region. ^1^H-^15^N-TROSY spectra of unbound DARC 1–60 (black) overlaid on ^1^H-^15^N-TROSY spectra of DARC 1–60 in the presence of excess unlabelled DBP-RII (red). Sequence assignments are shown for the unbound DARC ^1^H-^15^N-TROSY spectra. Peaks still visible in the presence of DBP-RII (red) are at DARC 1–60's N- and C- termini. Residues that disappear in the presence of DBP-RII are in the center of DARC and delineate the binding region.

DARC binds DBP-RII through its N-terminal 60 amino acid ectodomain [7] and DARC binding induces dimerization of DBP-RII [26]. Binding and dimer-induction *in vitro* has been demonstrated for non-tyrosine sulfated DARC N-terminal ectodomain with three cysteines mutated to alanine [26]. These cysteines in DARC are thus unlikely to be necessary for the interaction with DBP-RII but appear to be important to the recognition of chemokines, suggesting that disulfide linkages may be important for the folding of native DARC [27]. NMR experiments were performed to determine the region of an unsulfated version of the DARC N-terminal ectodomain contacted by DBP-RII (Fig. 1). Resonance assignments for the 60 N-terminal amino acids of DARC (DARC 1–60) were obtained by standard triple resonance experiments. The peaks are not well dispersed (Fig. 1) and chemical shifts closely match canonical random coil chemical shifts, consistent with a lack of secondary structure.

In the presence of DBP-RII, the large size of the 88 kDa DBP-RII∶DARC1–60 complex led to significant broadening of many peaks, preventing full assignment. However, comparison of the spectra between bound and unbound states revealed that signals corresponding to the first N-terminal 15–16 amino acids overlay well and have only modest line broadening. This result suggests that this region remained unstructured upon binding and does not directly contact DBP-RII. At the C-terminus, residues from 44–60 exhibit some line broadening or chemical shift changes, but this region, similar to that of the N-terminus, was still relatively unperturbed upon binding to DBP-RII. In contrast, peaks corresponding to the central region of DARC1–60 became significantly broadened, shifted, or disappeared in the bound complex. These results indicate that residues within the central region of the DARC ectodomain are highly perturbed upon interaction with DBP-RII and are thus most likely to directly contact DBP and form the minimal binding domain. This result is consistent with a region from DARC sufficient for blocking RBC binding by DBP-RII [22].

### The DARC ectodomain forms a helix that binds the dimer interface of DBP-RII

**Figure 2:**
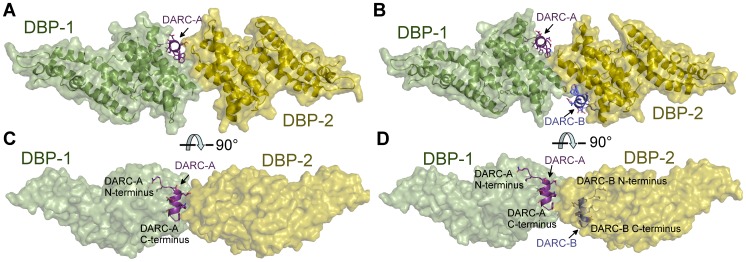
Crystal Structure of the DBP-RII∶DARC heterotrimer and heterotetramer. Overview of (**A**) DBP-RII∶DARC heterotrimer and (**B**) the DBP-RII∶DARC heterotetramer. Rotated views, (**C**) and (**D**), show DARC helices are oriented in parallel in the heterotetramer. DBP-RII monomers are in yellow and green. DARC monomers are in purple and blue.

Screening for crystallization conditions of DBP-RII in complex with DARC ectodomain constructs designed around the central binding region resulted in two crystal structures of the DBP-RII∶DARC complex. The first was a 1.95 Å crystal structure of a 2∶1 complex of DBP-RII∶DARC16–43 (Table 1). In this structure, two DBP-RIIs (DBP1 and DBP2) bind a single DARC (DARC A) creating the heterotrimer (Fig. 2A) with a total buried surface area of 2241.8 Å^2^. The second structure was a 2∶2 complex of DBP-RII∶DARC14–43 that was refined to 2.6 Å (Table 1). In the second structure, two DBP-RIIs each bind two DARCs (DARC A and DARC B) creating two DARC binding sites. This architecture creates a heterotetramer (Fig. 2B), with a total buried surface area of 3628.6 Å^2^. We postulated that the two structures represent snapshots in the assembly of the DBP-RII∶DARC complex and may define structural changes during step-wise binding. Additionally, in the heterotrimer, the second DARC binding site is preformed to accept another DARC. In both crystal forms the two DBP-RII molecules are not identical and no higher order symmetry exists in the asymmetric unit. Therefore, DARC binding results in two distinct DBP-RII molecules in each asymmetric unit.

**Table 1:** Data collection and refinement statistics.

	DBP-RII∶DARC heterotrimer	DBP-RII∶DARC heterotetramer
Data collection		
Space group	P2(1)	P1
Cell dimensions		
*a*, *b*, *c* (Å)	59.59, 66.99, 97.92	37.39, 59.47, 91.67
α, β, γ (°)	90, 102.112, 90	103.143, 91.098, 100.241
Resolution (Å)*	20-1.95 (2.0-1.95)	20-2.6 (2.7-2.6)
*R*_sym_ *	.098 (1.01)	0.052 (0.538)
*I*/σ*I* *	14.54 (2.11)	12.32 (1.69)
Completeness (%)*	99.8 (99.7)	95.2 (93.1)
Redundancy*	7.96 (7.96)	2.4 (2.2)
Refinement		
Resolution (Å)	20-1.95	20-2.6 Å
No. Reflections	55,044	22,121
*R*_work_/*R*_free_	16.65/20.15	18.29/23.28
No. Atoms (Non-Hydrogen)		
Protein	5,422	5,607
Ligand Organic	0	12
Water	462	52
*B*-factors		
Protein	38.86	70.62
Ligand Organic		74.64
Water	39.44	52.38
R.m.s. deviations		
Bond lengths (Å)	0.003	0.002
Bond angles (°)	0.743	0.543

Values in parentheses are for highest-resolution shell.

Data were collected on a single crystal for each dataset.

### DBP-RII interacts with DARC in a step-wise binding process

**Figure 3:**
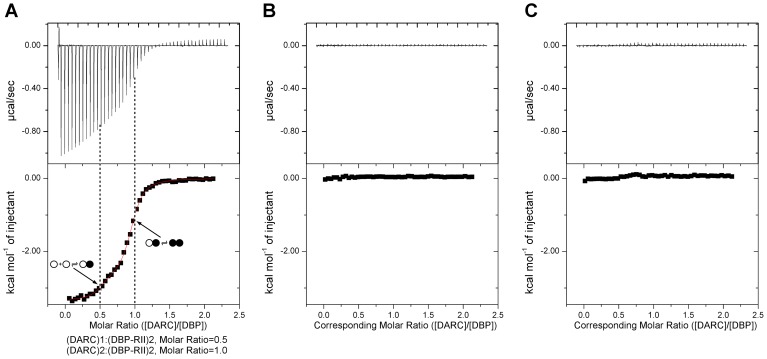
Isothermal titration calorimetry reveals step-wise binding of DARC to DBP-RII in solution. (**A**) A biphasic binding profile is observed indicating the formation of the heterotrimer at a molar ratio of 0.5 (*n_1_* = 0.44±0.02, *K_d1_* = 2183±125 nM, Δ*H_1_* = −2663±69 cal/mol) and heterotetramer at a molar ratio of 1 (*n_2_* = 0.50±0.02, *K_d2_* = 88.5±26.6 nM, Δ*H_2_* = −3338±23 cal/mol). The fit to the two independent site binding model is shown as a red line. Molar ratios are expressed as monomers of DBP-RII. Open circles denote unbound DBP, closed circles denote bound DBP. Titration of (**B**) PBS into DBP and (**C**) DARC into PBS reveals no observable profiles demonstrating the biphasic profile is due to DARC binding to DBP. In all cases, the top panel contains raw binding data, and the bottom panel changes in enthalpy associated with binding.

We utilized ITC to examine the mechanism of DBP-RII∶DARC engagement and assembly in solution. ITC is an excellent technique to unambiguously determine interaction stoichiometries and can be applied to examine step-wise and multi-state binding systems in solution. Titration of DARC1–60 into DBP-RII demonstrated that DARC binding occurs in a step wise assembly consistent with the crystallographic studies. A biphasic binding isotherm indicative of a two-state assembly was observed (Fig. 3). The first binding event has a molar ratio of 0.5, expressed in monomers of DBP-RII, indicative of a 2∶1 heterotrimeric complex of (DBP-RII)_2_∶(DARC1–60)_1_. The second binding event occurs at a molar ratio of 1 indicative of a 2∶2 heterotetrameric complex or (DBP-RII)_2_∶(DARC1–60)_2_. The data were fit to a two independent site binding model which suggested affinities of 2181±125 nM for the first binding event and 88.5±26.6 nM for the second binding event, consistent with high affinity binding. However, it should be noted that the two independent site binding model is not a perfect description of DBP-RII∶DARC binding as receptor-induced dimerization is not included in the model. Therefore, exact affinity determination will require further work necessary to define all thermodynamic parameters of binding. While the exact affinity will require a more detailed fitting model, the stoichiometries determined and thus the observation of step-wise assembly in solution are not affected by the fitting model selected and are reliable. In summary, the crystallographic and ITC solution data presented here demonstrate a multi-step sequential binding mechanism involving DARC-induced assembly of DBP-RII.

### Identification of molecular interactions between DARC and DBP-RII

**Figure 4:**
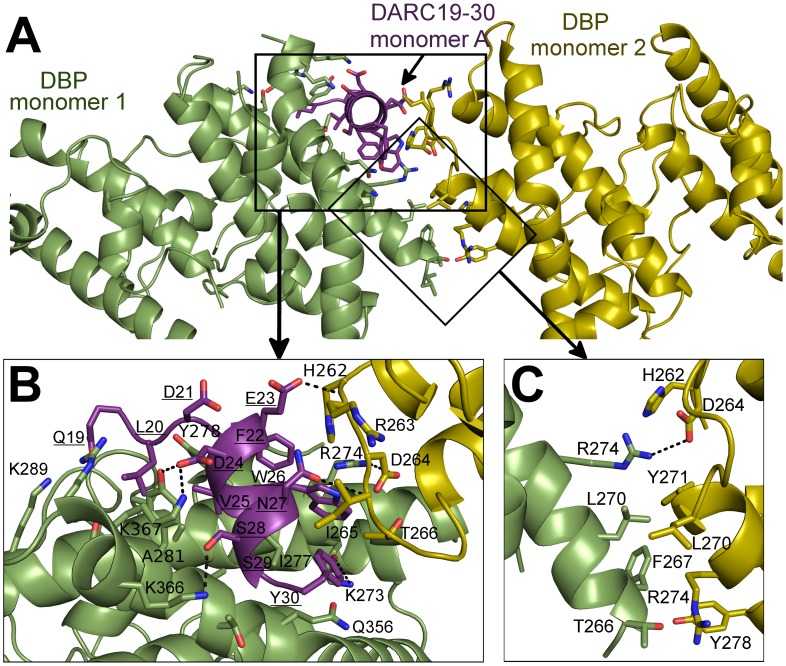
Binding interfaces of the DBP-RII∶DARC heterotrimer. (**A**) Global view of the DBP-RII∶DARC heterotrimer, showing (**B**) DARC monomer A interactions and (**C**) the DBP-RII homodimeric interface. DARC monomer A is in purple, DBP-RII monomer 1 is in green and DBP-RII monomer 2 is in yellow. Residue numbers are labeled and DARC residue labels are underlined.

**Figure 5:**
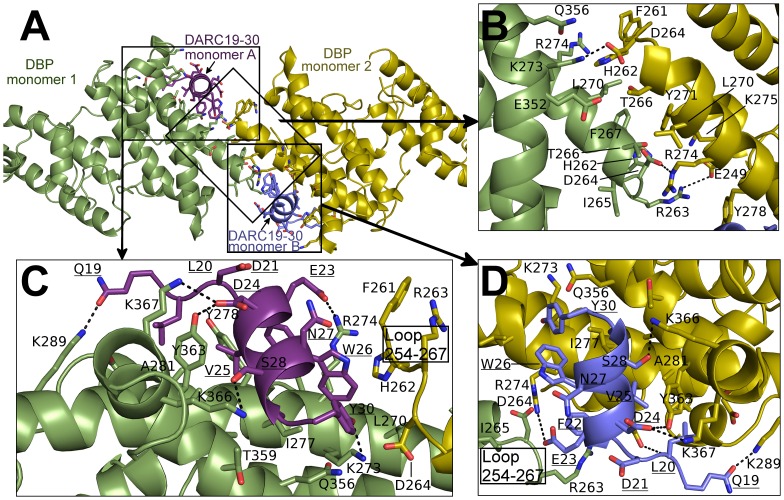
Binding interfaces of the DBP-RII∶DARC heterotetramer. (**A**) Global view of the DBP-RII∶DARC heterotetramer, showing (**B**) the DBP-RII homodimeric interface, (**C**) DARC monomer A interactions, and (**D**) DARC monomer B interactions. DARC monomer A is in purple, DARC monomer B is in blue, DBP-RII monomer 1 is in green and DBP-RII monomer 2 is in yellow. Residue numbers are labeled and DARC residue labels are underlined.

The NMR studies indicate DARC1–60 lacking three cysteines is unstructured in the absence of DBP-RII. In both structures, clear density is seen for DARC residues 19–30 (Fig. S1). DARC is induced to form an amphipathic helix in the crystal structures upon binding and engages a positively charged groove at the DBP-RII dimer interface (Fig. S1). All DARC interacting residues and the dimer interface of DBP-RII are located in subdomain 2 of DBP-RII. In addition to the dimer interface, each DARC binding site, one in the heterotrimer and two in the heterotetramer, can be broken into two interfaces: a primary DARC binding interface with one DBP-RII monomer, and a secondary DARC binding interface created by the second DBP-RII monomer (Fig. 4 and 5, Table 2 and 3). The DBP-RII homodimer interface is composed of DBP1 residues I265-R274 and DBP2 residues F261-Y278 in the heterotrimer (Fig. 4C), and DBP1 residues H262-R274 and DBP2 residues F261-Y278 in the heterotetramer (Fig. 5B). The primary DARC binding interface in both structures consists of DBP-RII residues L270-K289 of helix 4 and Q356-K367 of helix 7 (Fig. 4 and 5). DBP-RII binds the amphipathic DARC helices through a hydrophobic core flanked by electrostatic interactions.

**Table 2:** Heterotrimer interface residues determined by PDBePISA [47]: All residues in the interface are listed sequentially and do not indicate interacting pairs.

DARC binding site				Dimer Interface	
Primary DARC binding interface (DARC A to DBP 1)		Secondary DARC binding interface (DARC A to DBP2)			
DARC A	DBP 1	DARC A	DBP 2	DBP 1	DBP 2
GLN19	LEU270	PHE22	HIS262	PHE261	ILE265
LEU20	LYS273	GLU23	ARG263	HIS262	THR266
ASP21	ARG274	TRP26	ASP264	ASP264	PHE267
PHE22	ILE277	ASN27	ILE265	LEU270	LEU270
ASP24	TYR278	TYR30	THR266	TYR271	TYR271
VAL25	ALA281			ARG274	ARG274
TRP26	VAL282			LYS275	
SER28	ASP285			TYR278	
SER29	GLN356				
TYR30	THR359				
	ALA360				
	TYR363				
	SER364				
	LYS366				

**Table 3:** Heterotetramer interface residues determined by PDBePISA [47]: All residues in the interface are listed sequentially and do not indicate interacting pairs.

DARC binding site 1				DARC binding site 2				Dimer Interface	
Primary DARC binding interface (DARC A to DBP 1)		Secondary DARC binding interface (DARC A to DBP 2)		Primary DARC binding interface (DARC B to DBP 1)		Secondary DARC binding interface (DARC B to DBP 2)			
DARC A	DBP 1	DARC A	DBP 2	DARC B	DBP 1	DARC B	DBP 2	DBP 1	DBP 2
GLN19	GLU249	GLU23	PHE261	GLN19	LEU270	PHE22	ARG263	HIS262	GLU249
LEU20	LEU270	TRP26	HIS262	LEU20	LYS273	GLU23	ASP264	ARG263	PHE261
ASP21	LYS273	ASN27	ARG263	ASP21	ARG274		ILE265	ASP264	HIS262
PHE22	ARG274	TYR30	ASP264	PHE22	LYS275			ILE265	ASP264
GLU23	LYS275			GLU23	ILE277			THR266	THR266
ASP24	ILE277			ASP24	TYR278			PHE267	PHE267
VAL25	TYR278			VAL25	ALA281			LEU270	LEU270
TRP26	ALA281			TRP26	VAL282			TYR271	TYR271
SER28	VAL282			SER28	ASP285			LYS273	ARG274
SER29	ASP285			SER29	LYS289			ARG274	LYS275
TYR30	LYS289			TYR30	GLN356			GLU352	TYR278
	GLN356				THR359			GLN356	
	THR359				ALA360				
	ALA360				TYR363				
	TYR363				SER364				
	SER364				LYS366				
	LYS366				LYS367				
	LYS367								

The secondary DARC binding interface is formed by residues V254 to F267 (loop 254–267) (Fig. 4B, 5C–D). When DBP-RII is not bound to DARC this region is disordered [26]. Loop 254–267 contains residues which are required for binding [20], and are recognized by neutralizing antibodies [17]. When DARC is bound, loop 254–267 becomes ordered and engages the DARC bound by the primary interface of a neighboring DBP-RII. In the heterotrimer, residues H262-T266 make contacts at the secondary interface (Fig. 4B). In the heterotetramer, DBP1 residues R263-I265 and DBP2 residues F261-D264 contact DARC of the opposing monomer (Fig. 5C–D). Thus, DARC is sandwiched between two DBP-RII molecules in each DARC binding site.

### Architectural transitions upon receptor binding

In the absence of receptor, DBP-RII crystallized as a dimer stabilized by phosphates [26]. Although this prior structure resembles the receptor-bound conformation presented here, there are substantial structural differences in the architecture of the dimer compared to the heterotrimer or heterotetramer. These differences are crucial towards correctly defining the invasion interaction. In the heterotrimer structure, a new DBP-RII homodimer interface is created by a translation of 7 residues covering 12 Å along helix 4, relative to the unbound structure (Fig. S2A). The heterotetramer structure has a larger translation along the same interface in the same direction, with a second 12 Å displacement relative to the heterotrimer (Fig. S2B), and a 23 Å displacement relative to the unbound DBP-RII homodimer interface (Fig. S2C). The directionality of these transitions is consistent with sequential steps in a stepwise mechanism of receptor binding. While there are major changes in the DBP-RII∶DARC complex architectures, individually each DBL domain aligns well to the DBP-RII DBLs solved in the absence of receptor (Fig. S2D–G).

### Critical contacts in DARC

The observation that DARC 19–30 is contacted by DBP-RII is consistent with alanine scanning work [23]. Mutation of DARC residues 20–22 and 24–26 abrogated binding in a direct protein interaction assay. Each of these residues, with the exception of D21, makes direct contacts with DBP-RII and are buried in the complex (Fig. 4 and 5). D21, which is required for DBP-RII binding but does not directly contact DBP-RII, stabilizes the DARC N-terminal helix dipole by positioning its acidic side chain directly over the helix. E23, on the other hand, is on the surface of the complex and is solvent exposed. Mutation of E23 to alanine had no effect on binding consistent with its location in the complex.

Sulfotyrosine residues in DARC increase DARC's ability to inhibit DBP-RII RBC binding [19]. A previous structure of DBP-RII alone identified a potential sulfotyrosine binding site that includes residues K273 and Q356 [26]. In the receptor-bound structure presented here, the hydroxyl of DARC Y30 points directly at this pocket created by K273, and Q356 (Fig. S3). Therefore, sulfotyrosine 30 appears to bind at this site when DARC is sulfated. DARC residues 14–43 were included in our crystallographic studies. However, clear density was only observed for residues Q19 to Y30, and no density was present for the remainder of DARC. The crystallographic data along with the RBC binding studies discussed above support that residues 19–30 constitute a critical interaction site with DBP-RII.

### Residues in DBP-RII that contact DARC are required for RBC engagement

**Figure 6:**
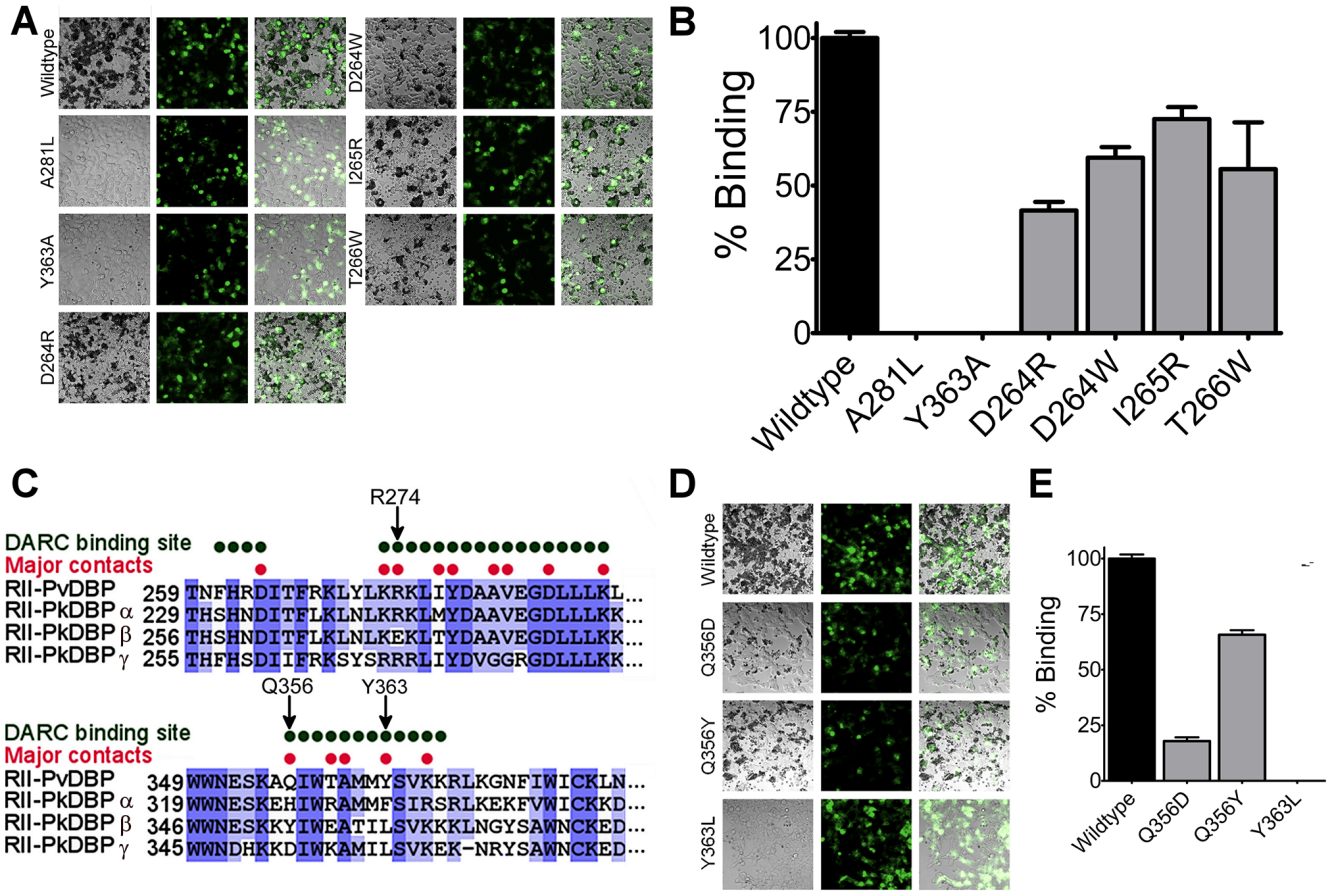
The structural studies define red blood cell binding. (**A**) Adherent HEK293 cells in grey bind to darker, smaller red blood cells when transfected with a DBP-RII surface expression plasmid with a GFP marker. Red blood cell rosetting images for DBP-RII mutants, showing bright field (left), GFP (center), and merged images (right). (**B**) Percentage of cells expressing point mutants which bind red blood cells, relative to wildtype, shown with standard error. (**C**) The major DBP-RII∶DARC residues identified in the crystal structures are indicated by red dots. Non-conservative *P. knowlesi* mutations at critical DBP-RII∶DARC contact residues 274, 356, and 363 suggest why PkDBPα but not PkDBPβ or PkDBPγ bind DARC. (**D**) Red blood cell rosetting images for DBP-RII receptor specificity mutants, showing bright field (left), GFP (center), and merged images (right). (**E**) Percentage of cells expressing receptor specificity point mutants which bind red blood cells, relative to wildtype, shown with standard error.

Having identified residues of DBP-RII that directly contact DARC, structure-guided mutagenesis was used to determine whether this model of binding explains DBP engagement of RBCs (Fig. 6). Mutation of residues Y363 or A281 in the primary DARC binding interface led to a complete loss of binding in a functional RBC binding assay. This is expected as DARC binding drives complex formation and mutations preventing DARC binding will completely abrogate complex formation and attachment. These results are consistent with previous mutational studies that identified potential interaction residues between DBP-RII and DARC [20], [21], several of which map to the interaction surfaces identified here. Residues D264, I265 and T266 are located in the secondary DARC binding interface and directly contact DARC. Mutation of these residues resulted in a loss in RBC binding, demonstrating that the secondary DARC binding interface plays a role in engaging DARC during RBC invasion. Large bulky amino acid changes are required in order to disrupt binding by mutation at the secondary binding site, as is expected from the large contact area created by the additional interfaces in the full complex. The need for large changes is consistent with a lack of an effect on binding when mutations to alanine or conservative changes were introduced at these residues [20], [28]. Together, these results support the functional role of both DARC binding interfaces.

DARC on erythrocytes is putatively sulfated [19]. The mutational studies discussed above show that sulfation of DARC on erythrocytes, and the remaining segments of full length DARC, cannot compensate for the loss of the critical binding sites containing L270-K289 and Q356-K367 that bind DARC 19–30. In particular, recombinant DBP-RII containing Y363A is unable to bind to sulfated DARC *in vitro* [21]. This result also demonstrates that sulfation of DARC cannot compensate for the loss of the DARC binding sites induced by the Y363A mutation. Together, the results identify essential binding residues within DBP-RII, consistent with prior studies, which form critical binding sites required for engagement of DARC.

### DBP receptor specificity is manifested through changes in the DARC binding sites

*P. knowlesi* uses three different DBP homologs to invade human and rhesus macaque RBCs [10]. Only *P. knowlesi* DBPα (PkDBPα) binds DARC, while PkDBPβ and PkDBPγ do not bind human RBCs and recognize a receptor other than DARC [10]. This receptor specificity is likely due to three amino acid changes in the critical DARC binding site of DBP that are changed to non-conservative amino acids in PkDBPβ and/or PkDBPγ (Fig. 6C). The DBP residue with the most extensive DARC contacts is Y363. In both PkDBPβ and PkDBPγ, Y363 is changed to leucine. Mutation of Y363L resulted in a complete loss of binding (Fig. 6D and E) consistent with a role for this residue in receptor specificity. Additionally, R274E and Q356Y in PkDBPβ, as well as Q356D in PkDBPγ, would likely destabilize DARC binding as both R274 and Q356 contact the DARC residues E23 and Y30, respectively. It has been demonstrated that a R274E mutation in DBP-RII completely prevents RBC binding [26]. Mutation of Q356D and Q356Y resulted in a loss in RBC binding, with Q356D having a large effect (Fig. 6D and E). Thus, contacts identified in the DBP∶DARC structure provide insight into why PkDBPβ or PkDBPγ do not bind DARC on human RBCs.

## Discussion

Numerous functional and immunological studies have been conducted on the *P. vivax* DBP invasion system since the Duffy antigen was found to be essential to this species in the 1970's [5]. Here we have shown using crystallography that DBP-RII binds DARC forming a heterotrimer and heterotetramer and demonstrated using ITC that the interaction assembles in discrete steps. In addition, we identify DARC residues 19–30 as a critical interaction site for DBP-RII binding, and thus to tight junction formation during invasion. These studies also identify DBP-RII residues that directly contact the DARC receptor, including L270-K289, Q356-K367 and F261-T266. The structural and mechanistic basis of Duffy recognition by *P. vivax* provides a context for prior work and may assist with the rational design of therapeutics and vaccines targeting this essential *P. vivax* binding interaction.

**Figure 7:**
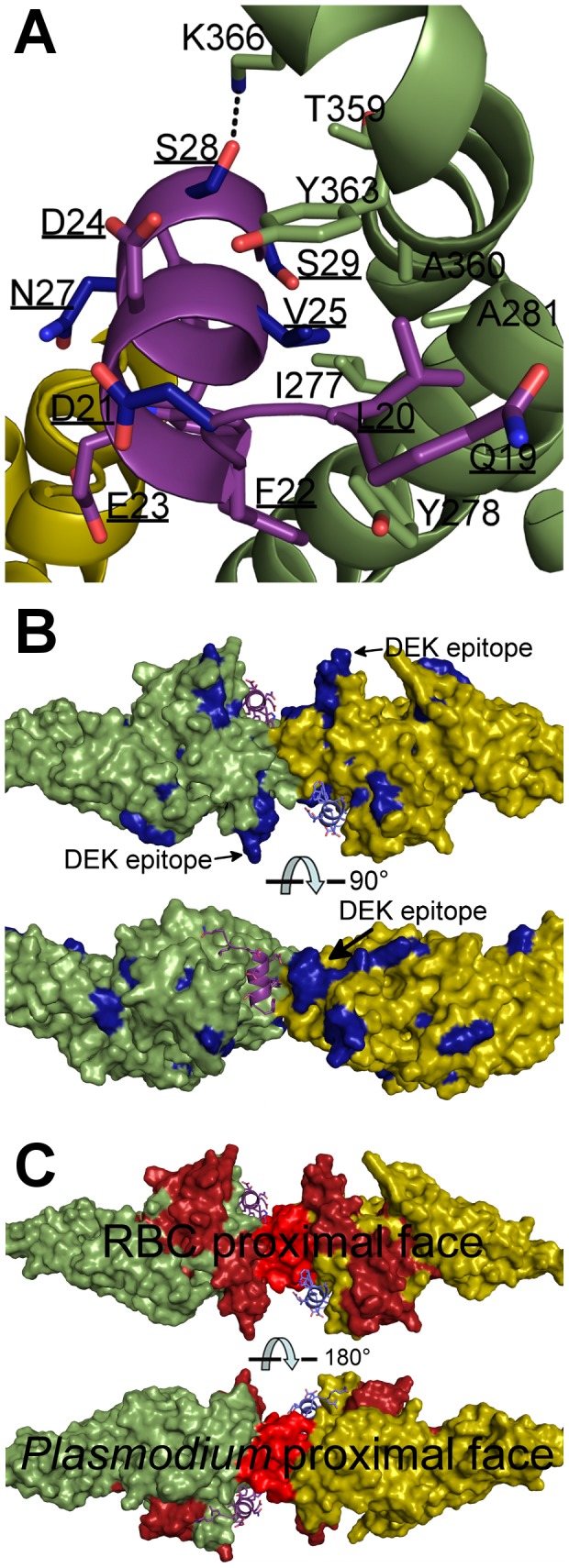
Mapping polymorphic residues and inhibitory epitopes reveals targets of selective pressure. DBP-RII molecules are in green and yellow. DARC molecules are in purple and blue. DARC residue labels are underlined. (**A**) Nonsynonymous DARC polymorphisms in primates, residues colored in blue, which make critical contacts with DBP-RII provide a mechanism for inter-species transmission barriers. (**B**) Polymorphic DBP residues, in blue, are spread throughout the molecule. The most polymorphic region of DBP is the “DEK epitope” opposite the DARC14–43 binding site. (**C**) Inhibitory epitopes, in red and brown, map to the heterotetramer interface, DARC binding pockets and RBC proximal face of DBP-RII.

Phylogenetic studies have identified primate DARC residues under positive selection to block *Plasmodium* infection [29]. V25 in DARC is especially polymorphic among primates and under strong positive selection. This is because DBP makes essential hydrophobic packing interactions with V25 (Fig. 7A), disruption of which would strongly destabilize binding. Gorillas, the ancient host of *P. falciparum* [30], have a V25A mutation in DARC. This disrupts hydrophobic interactions, prevents DBP binding [23], and serves as an inter-species barrier to *P. vivax* infection.

Sequencing of parasite populations show particular sites of DBP are under strong positive selective pressure to evade the immune response [31]–[33]. Many polymorphic DBP residues are located far from the DARC binding sites (Fig. 7B) [20], [21]. The most polymorphic region of DBP, the DEK epitope, forms a ridge directly opposite DARC, flanking the secondary binding interface and homodimer interface. Converting this epitope to small, nonpolar amino acids, focuses the immune response towards cross-specific neutralizing epitopes [34]. Our results suggest that this hypervariant DEK epitope does not play a direct role in DARC binding. Polymorphisms in the DEK epitope should not affect DBP function, but could interfere with immune recognition of DBP. Antibody recognition of the hypervariant DEK epitope may neutralize *P. vivax* by preventing assembly of the DBP-RII∶DARC complex, and thus sterically preventing DBP-RII homodimeric contacts. Polymorphisms are heavily selected for within the DEK epitope suggesting *P. vivax* evades this potentially neutralizing antibody response by antigenic variation within these residues.

**Figure 8:**
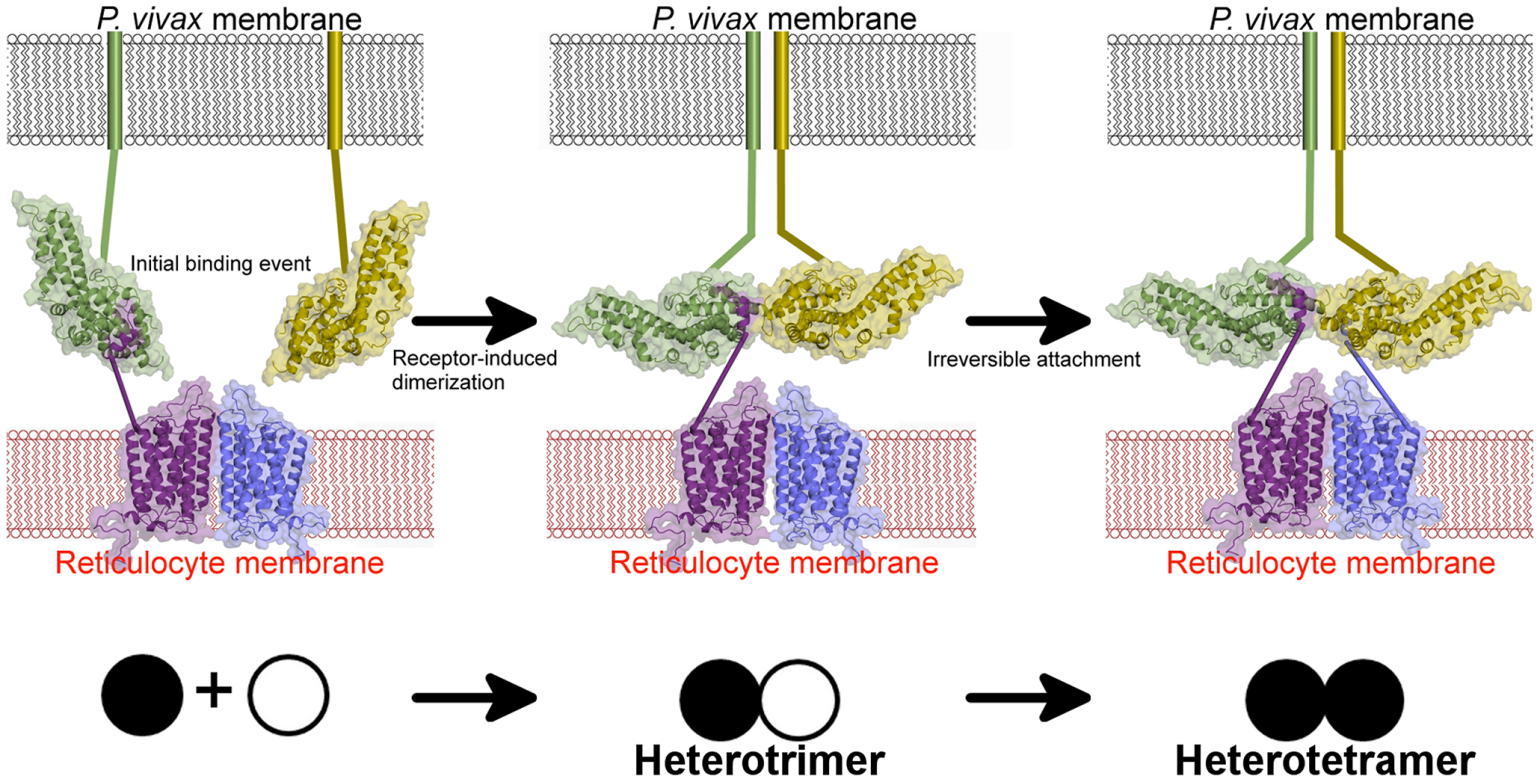
A model for attachment during invasion. An initial binding event is followed by receptor-induced dimerization, as in the DBP-RII∶DARC heterotrimer. This brings a second DBP-RII molecule in close proximity to a second DARC ectodomain in the DARC homodimer. A second binding event creates the DBP-RII∶DARC heterotetramer. DBP-RII molecules are in green and yellow and DARC19–30 molecules are in purple and blue. The DARC homodimer is represented by a homology model. A schematic for the stepwise assembly is shown at the bottom. Closed circle – bound DBP-RII, open circle – unbound DBP-RII.

The studies presented here define a putative mechanism for known neutralizing epitopes of *P. vivax* DBP-RII and illuminate potential new targets for naturally acquired immunity. Specifically, both DARC helices are oriented in a parallel manner (Fig. 2B and 2D), and DARC itself is a homodimeric GPCR. Because only 30 amino acids, DARC residues G31 to S60, separate the structure from the RBC membrane, the surface of the DBP-RII dimer with DARC Y30 is proximal to the RBC membrane, and the alternate surface faces the *Plasmodium* membrane. Antibody recognition of DBP-RII's RBC proximal surface prior to DARC binding neutralizes DBP by sterically preventing DBP-RII from approaching the RBC surface. This model is confirmed by recent work which identified several DBP neutralizing epitopes recognized by human antibodies from individuals living in endemic areas [17] which are located either directly at the DARC binding sites or at DBP-RII's RBC proximal surface (Fig. 7C). In addition, the most potent known neutralizing epitope for DBP includes much of helix 4 and loop 254–267 [17], which contains the DBP-RII dimerization interface and the DARC binding sites. Disrupting DBP-RII dimerization would both destabilize DARC binding by preventing secondary DBP-RII contacts and destroying interaction contributions due to avidity. Recently, mouse monoclonal antibodies that bound subdomain 3 of DBP-RII also blocked binding to erythrocytes [35]. Subdomain 3 lies in close proximity to the RBC surface (Fig. 8) and these antibodies may block binding by preventing DBP-RII from approaching the RBC surface and contacting DARC. The identification of two DARC binding sites within DBP-RII and the structural orientation of each molecule provide insight into the mechanisms of antibody inhibition of *P. vivax* RBC invasion. It appears that antibodies targeting DBP-RII are capable of preventing DARC binding by recognizing DBP-RII's RBC proximal surface, DARC recognition sites, or the homodimeric interface, and may block invasion using other, currently unidentified mechanisms.

The crystallographic and ITC solution studies presented here support a step-wise binding model in which receptor-induced DBP-RII dimerization facilitates formation of a heterotrimer that subsequently recruits a second DARC molecule to form a heterotetramer (Fig. 8). Due to avidity contributions to binding inherent in a two-site mechanism, this heterotetrameric complex may enable the observed tight binding of *P. vivax* to the RBC membrane. Since recombinant DBP-RII is monomeric in the absence of DARC when examined in solution [26], the dimer interface and DARC binding pockets are exposed and accessible to antibodies prior to DARC engagement. DARC is known to exist as a homodimeric and heterodimeric GPCR [36]. The heterotrimer and heterotetramer could represent DBP-RII binding to a DARC heterodimer or homodimer, respectively. The observed transitions may be a selectivity mechanism for DBP-RII to preferentially bind homodimeric DARC while maintaining the ability to bind to a DARC heterodimer. The binding model proposed here is applicable to other DBL domain proteins that may oligomerize upon receptor binding [9], [26], [37]–[39]. Since dimerization is prevalent in receptor signaling, it is plausible that complex assembly initiates a signal through the transmembrane and cytoplasmic domains of DBP to activate pathways of invasion.

Although structure determination of the DBP-RII∶DARC complexes allows for visualization and identification of critical contact points, the relevance of each intermediate to complex assembly in solution is not immediately known from the static pictures of binding. To begin to assess the biological role of complex assembly, we utilized ITC to demonstrate that two binding events corresponding to the formation of a heterotrimer and heterotetramer exist in solution. We further tested mutant DBP-RII constructs for RBC binding and demonstrated that these mutations ablate binding to RBCs, supporting the biological role of the DARC contacts identified here as well as the role of the dimer interface. In addition, the large buried surface area for both DARC binding sites and the ITC measurements suggest high affinity interactions. This study thus unambiguously identifies DARC residues 19–30 as a critical binding element that interacts with DBP residues L270-K289, Q356-K367 and F261-T266.

The biphasic profile obtained by ITC is different from studies previously reported where a single binding event with a molar ratio of 1 was observed indicative of the heterotetramer [26]. This difference is likely due to the buffer conditions used in each case. In prior studies, titrations were performed at a salt concentration of 50 mM while the studies presented here were performed in PBS to examine binding under physiological conditions. These results suggest that observation of the heterotrimer intermediary step by ITC is salt dependent. Never-the-less, the biphasic profile and step wise binding mechanism presented here are representative of the assembly mechanism under physiologically relevant conditions.

In both crystal structures, clear electron density was observed for residues 19–30 of DARC. The binding pockets in DBP identified here are distinct from a patch of residues previously suggested to engage DARC [40] (Fig. S4). These residues were proposed based on loss of binding of DBP to DARC upon mutation; however, no data for direct interaction of this patch of residues with DARC was presented. In contrast, the crystal structure of *P. vivax* DBP in complex with DARC demonstrates clear contact points between the two binding partners, and mutagenesis data strongly supports the critical role of the residues identified in the binding pockets.

Mutational studies [20], [21] and glycan shielding experiments [28] have identified several patches of residues that affect binding of DBP to RBCs, some of which overlap and are consistent with the DARC contacts identified here. There are additional residues outside the DARC binding pockets that when altered reduce binding [20], [21], [28]. Therefore, the complete range of interactions between DARC and DBP-RII will likely include additional patches of residues in DBP. Specifically, sulfation of tyrosine 41 and the Fya/Fyb polymorphism have been shown to play a role in binding [18], [19]. However, the specific mechanism by which these changes impact binding is unknown. In addition, an association between the N-terminus and additional extracellular loops of DARC has been suggested to play a role in chemokine binding to DARC [27]. Further studies are necessary to fully understand the role of tyrosine sulfation, the Fya/Fyb polymorphism, and the potential role of additional regions and loops of DARC during *P. vivax* binding and RBC invasion.

The identification of critical DARC binding pockets presented in this study may facilitate the rational design of therapeutics that seek to inhibit RBC binding. Small molecule inhibitors that bind to the DARC binding pocket and prevent DARC engagement could disrupt merozoite invasion, and thus *P. vivax* growth. Alternately, disruption of complex assembly by small molecules, as has been described for AMA-1:RON-2 [41], or antibodies would also provide novel methods for preventing RBC invasion. Since complex assembly is dependent on DARC binding, the most potent disruption of RBC engagement is expected by targeting DARC binding sites. Recent work examining the mechanism of monoclonal antibodies targeting EBL ligands supports the view that targeting receptor binding sites and multimerization interfaces of EBL ligands effectively prevents RBC binding and limits parasite growth *in vitro* [42]. Additionally, glycan masking experiments with DBP-RII identified the dimer interface and surfaces adjacent to this interface as critical binding sites and targets of an inhibitory antibody response [28]. This result supports the importance of identifying and targeting essential functional residues/interfaces of DBP-RII and confirms the biological importance of the contact points identified in this study.

This work also has implications for diagnostics and measures aiming to quantify the immune response to natural infection and in determining the efficacy of vaccine candidates. For a more robust measure of protection, these approaches should quantify the immune response to the functional regions identified here in addition to the response to the entire DBP-RII DBL domain. This study thus expands our understanding of the essential interaction between DBP and DARC and may aid in defining *in vivo* studies that seek to examine the extensive receptor-ligand binding interactions that are essential to RBC invasion by *Plasmodium* species.

## Materials and Methods

### Protein expression, purification, and complex formation

DBP-RII and DARC were produced as previously described [26]. DARC constructs were expressed in *E. coli* with an N-terminal GB1 tag, followed by a hexahistidine tag and a PreScission Protease cleavage site. Nickel-NTA chromatography followed by PreScission protease treatment and gel filtration resulted in a homogenous sample.

### NMR

NMR data were collected at 298 K on a 600 MHz Bruker spectrometer equipped with a triple-resonance room temperature probe and a QCI cryoprobe. Backbone assignments for the non-proline residues in DARC 1–60 were obtained using standard HNCACB, CBCA(CO)NH, HN(CA)CO, and HNCO experiments. Once the DARC 1–60 backbone resonances had been assigned, we collected 1H-15N-TROSY spectra of DARC 1–60 in the presence of DBP-RII. As DARC residues tightly bound to DBP-RII in a large complex are not visible, peaks which remain in the DBP-RII∶DARC 2D 1H-15N TROSY and 3D TROSY triple resonance spectra revealed residues which are not bound by DBP-RII.

### Crystallization and data collection

Before complex formation, DBP-RII and DARC were purified separately by size-exclusion chromatography to remove any trace aggregates in either sample. The DBP-RII∶DARC complexes were prepared by mixing purified DBP-RII and purified DARC in 1∶1.2 molar ratio. The DBP-RII∶DARC complexes were purified using size-exclusion chromatography in 20 mM 4-(2-hydroxyethyl)-1-piperazineethanesulfonic acid (HEPES), pH 7.4, and 50 mM sodium chloride. These sample was then concentrated in an Amicon concentrator with a 3-kDa molecular weight cutoff to 20 mg ml^−1^ for crystallization trials.

Native DBP-RII∶DARC crystals of both constructs were grown by hanging-drop vapor diffusion by mixing 1 µl of protein solution at 20 mg ml^−1^ and 1 µl of reservoir solution containing 0.1 M HEPES pH 7.4 and 20% (w/v) polyethylene glycol 6000. Crystallization of DBP-RII in complex with DARC 16–43 yielded the heterotrimeric structures, while crystallization of DBP-RII in complex with DARC 14–43 yielded the heterotetrameric crystals. The different crystal forms are not due to the DARC constructs used, rather serendipitous formation of one or either of the stable states upon complex assembly. Crystals were cryoprotected by transfer to reservoir solutions supplemented with glycerol and flash frozen in liquid nitrogen. Data for the heterotetramer was collected at a wavelength of 1.0 Å at beamline 4.2.2 of the Advanced Light Source, Lawrence Berkeley National Laboratory. Data for the heterotrimer was collected at a wavelength of 0.97929 Å at beamline 19-ID of the Advanced Photon Source, Argonne National Laboratory. Data reduction and processing was performed in XDS [43]. Data collection statistics are shown in Table 1.

### Structure solution

Both structures were solved by molecular replacement using DBP-RII apo-structure [26] leading to a starting model with Rwork/Rfree of 37.3%/38.1% for the heterotrimer and an Rwork/Rfree of 30.0%/30.1% for the heterotetramer. NCS restraints were not imposed on the two copies of DBP-RII during refinement as it was clear from electron density maps they were not identical. Subsequent automated rebuilding in PHENIX AutoBuild [44], manual rebuilding in COOT0.7 [45] and refinement in PHENIX1.7.3 [44] lead to a final model with Rwork/Rfree of 16.62%/20.47% for the heterotrimer and 18.29%/23.28% for the heterotetramer (Table 1). These low R factors combined with the good Ramachandran plot statistics analyzed by MolProbity [46] indicated that structure refinement was complete. Residue distributions in the Ramachandran plot for the heterotrimer were 98.42% allowed, 1.58% additionally allowed and 0% disallowed. Residue distributions in the Ramachandran plot for the heterotetramer were 95.72% allowed, 4.28% additionally allowed and 0% disallowed. The atomic coordinates and structure factors for the structure have been deposited in the protein data bank with accession numbers 4NUU and 4NUV.

### Isothermal titration calorimetry

DBP-RII was prepared as described above, with the addition of an ion-exchange chromatography step prior to ITC measurements. DBP-RII and DARC1–60 were exchanged into PBS to ensure measurements were made under physiological conditions. ITC experiments were carried out at 10°C using a VP-ITC instrument (MicroCal). DARC1–60 at 1.3 mM was titrated into 1.4 mL of 130 µM DBP-RII. For control experiments, 1.3 mM DARC 1–60 was titrated into 1.4 mL of PBS, and separately PBS was titrated into 1.4 mL of 130 µM DBP-RII. Traces were analyzed using Origin Version 5.0 (MicroCal). Stoichiometry and binding constants were calculated by fitting the integrated data to an independent two-site binding model after a double subtraction of both controls from the experimental titration. Protein concentrations were determined by absorbance measurements under denaturing conditions (6 M guanidinium hydrochloride, 10 mM dithiothreitol).

### Functional studies

DBP-RII with a C-terminally fused green fluorescent protein (GFP) was cloned into plasmid pRE4 for surface expression in mammalian cells. Single-amino-acid mutations were introduced in DBP-RII using the QuickChange method (Stratagene). Fresh monolayers of HEK293T cells were cultured in 3.5-cm-diameter wells and transfected with 2 µg ml^−1^ DNA in polyethyleneimine. The binding assay was performed 20 h after transfection. Anonymized human RBCs (ZenBio) were added to each well in a 10% suspension, incubated at 37°C for 1 h, and washed three times with PBS. Binding was quantified by counting rosettes observed over ten fields of view at ×20 magnification. Transfected HEK 293T cells with five or more attached RBCs were defined as positive rosettes. In each experiment, three wells of HEK 293T cells were transfected for each mutation. Cell counting was performed using ImageJ (NIH) on randomized images. Three fields of view from ten independent transfections (final n = 30) were counted for each sample (wild type or mutant). Significance was tested by a paired two-tailed Student's t-test as the data were normally distributed and had large sample sizes (n = 30).

## Supporting Information

Figure S1 DARC residues 19–30 are contacted by DBP-RII. DARC19–30 binds to a positively charged groove at the DBP-RII dimer interface of both (**A**) the DBP-RII∶DARC heterotrimer and (**B**) the DBP-RII∶DARC heterotetramer. Electrostatic potential is shown from −7.5 to 7.5 kT/e with positive potential in blue and negative potential in red. 2fo-fc electron density maps, contoured at 1σ clearly show the presence of (**C**) a single DARC19–30 in the heterotrimer and both (**D**) DARC19-30A and (**E**) DARC19-30B in the two DBP-RII binding sites of the heterotetramer. DARC monomers are in purple and blue and DBP-RII monomers are in green and yellow.
(TIFF)

Figure S2 Upon receptor binding, new regions of DBP-RII become structured, while preexisting structural regions undergo no major conformational changes. During the transition from the heterotrimeric to heterotetrameric complex, a change in the overall architecture of the DBP-RII dimer is observed. In (A–C) the DARC-bound DBP-RII heterotetramer is green and yellow, the DARC-bound DBP-RII heterotrimer is light green and light yellow, and unbound DBP-RII is dark green and orange. Structural transitions in each case are designated with an arrow as well as with the distance of the structural shift. (**A**) A translation covering 12 Å along helix 4 defines the difference between the heterotrimeric structure and a prior structure of DBP-RII in the absence of receptor. (**B**) A translation covering 12 Å across helix 4 is the difference between the heterotrimeric structure and the heterotetrameric structure. (**C**) A translation covering 23 Å along helix 4 is the difference between the heterotetrameric structure and DBP-RII in the absence of receptor, which defines the full shift following binding of both DARC molecules. (**D–G**) Alignments of the individual monomers of the DBP-RII∶DARC heterotetramer and unbound DBP-RII. (**D**) Monomer A of the heterotetramer (green) with monomer A unbound (dark green), (**E**) monomer A of the heterotetramer (green) with monomer B (orange) unbound, (**F**) monomer B of the heterotetramer (yellow) with monomer A unbound (dark green), (**G**) monomer B (yellow) of the heterotetramer with monomer B unbound (orange).
(TIFF)

Figure S3 The sulfotyrosine binding site. DBP-RII molecules are in green and yellow. The bound DARC molecule is shown in purple. (**A**) Phosphate or selenate in the apo DBP-RII structure occupy the same position as (**B**) DARC Y30, defining the sulfotyrosine binding pocket.
(TIFF)

Figure S4 The DARC binding pockets are distinct from residues previously suggested to bind DARC from mutagenesis studies. DBP-RII monomers are in yellow and green. DARC monomers are in purple and blue. Residues previously suggested [40] to contact DARC are in black.
(TIFF)
