# Supplementary figures and images for: Red Blood Cell Invasion by Plasmodium vivax: Structural Basis for DBP Engagement of DARC
Source: PLoS Pathog. 2014 Jan 9;10(1):e1003869. doi: 10.1371/journal.ppat.1003869 (PMC3887093; doi:10.1371/journal.ppat.1003869)

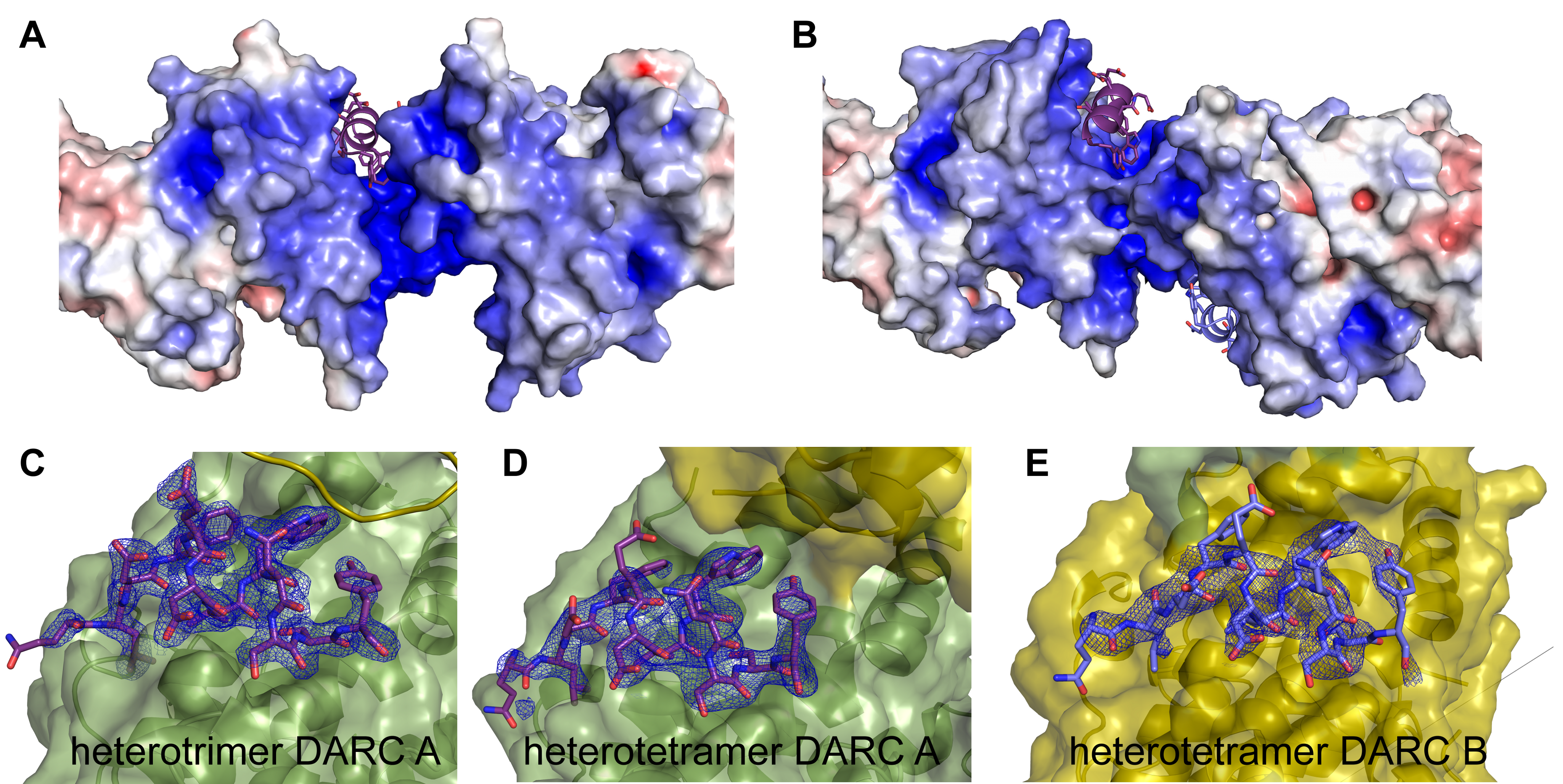

Supplement: Figure S1 — DARC residues 19–30 are contacted by DBP-RII. DARC19–30 binds to a positively charged groove at the DBP-RII dimer interface of both (A) the DBP-RII∶DARC heterotrimer and (B) the DBP-RII∶DARC heterotetramer. Electrostatic potential is shown from −7.5 to 7.5 kT/e with positive potential in blue and negative potential in red. 2fo-fc electron density maps, contoured at 1σ clearly show the presence of (C) a single DARC19–30 in the heterotrimer and both (D) DARC19-30A and (E) DARC19-30B in the two DBP-RII binding sites of the heterotetramer. DARC monomers are in purple and blue and DBP-RII monomers are in green and yellow. (TIFF) [file ppat.1003869.s001.tiff]

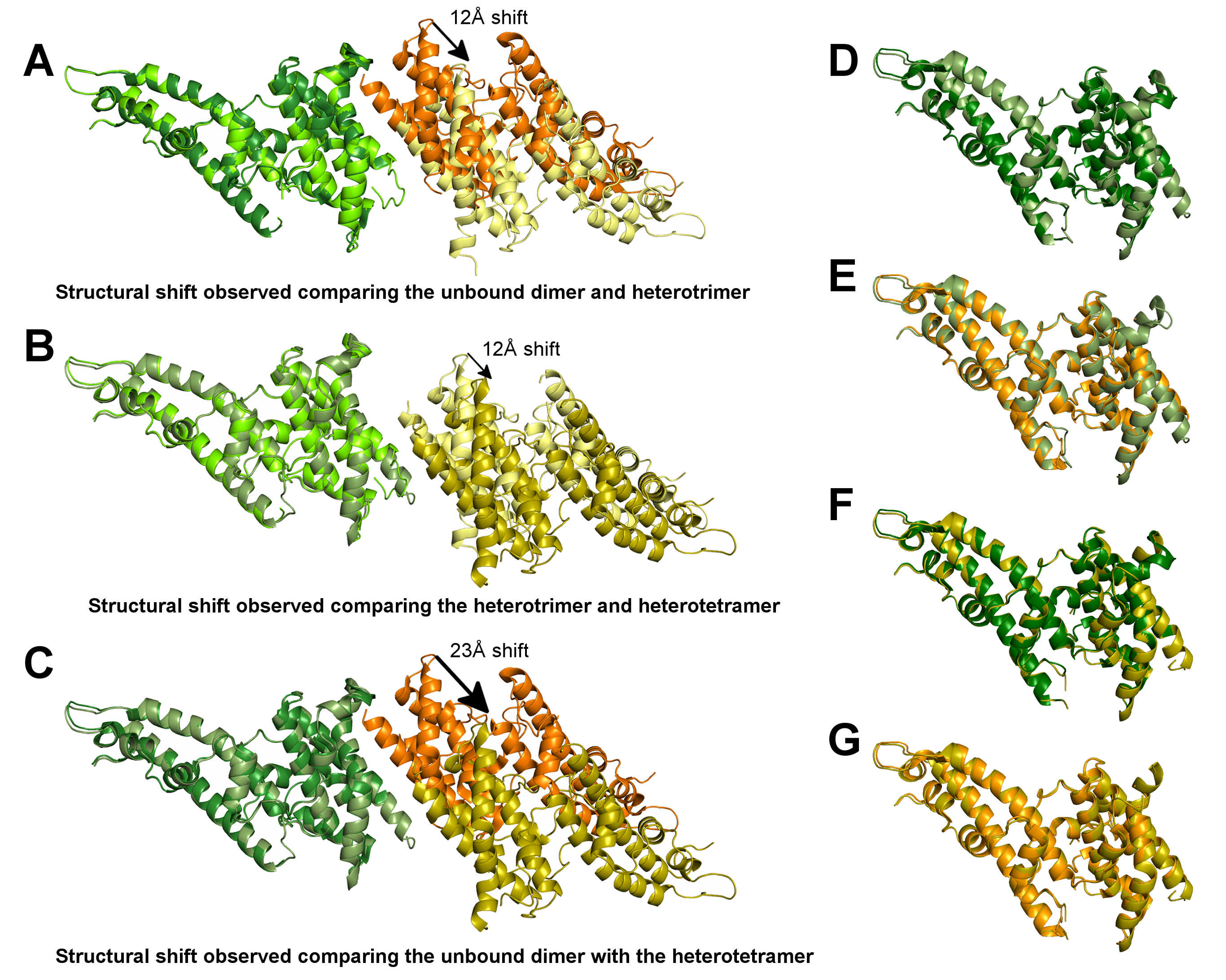

Supplement: Figure S2 — Upon receptor binding, new regions of DBP-RII become structured, while preexisting structural regions undergo no major conformational changes. During the transition from the heterotrimeric to heterotetrameric complex, a change in the overall architecture of the DBP-RII dimer is observed. In (A–C) the DARC-bound DBP-RII heterotetramer is green and yellow, the DARC-bound DBP-RII heterotrimer is light green and light yellow, and unbound DBP-RII is dark green and orange. Structural transitions in each case are designated with an arrow as well as with the distance of the structural shift. (A) A translation covering 12 Å along helix 4 defines the difference between the heterotrimeric structure and a prior structure of DBP-RII in the absence of receptor. (B) A translation covering 12 Å across helix 4 is the difference between the heterotrimeric structure and the heterotetrameric structure. (C) A translation covering 23 Å along helix 4 is the difference between the heterotetrameric structure and DBP-RII in the absence of receptor, which defines the full shift following binding of both DARC molecules. (D–G) Alignments of the individual monomers of the DBP-RII∶DARC heterotetramer and unbound DBP-RII. (D) Monomer A of the heterotetramer (green) with monomer A unbound (dark green), (E) monomer A of the heterotetramer (green) with monomer B (orange) unbound, (F) monomer B of the heterotetramer (yellow) with monomer A unbound (dark green), (G) monomer B (yellow) of the heterotetramer with monomer B unbound (orange). (TIFF) [file ppat.1003869.s002.tiff]

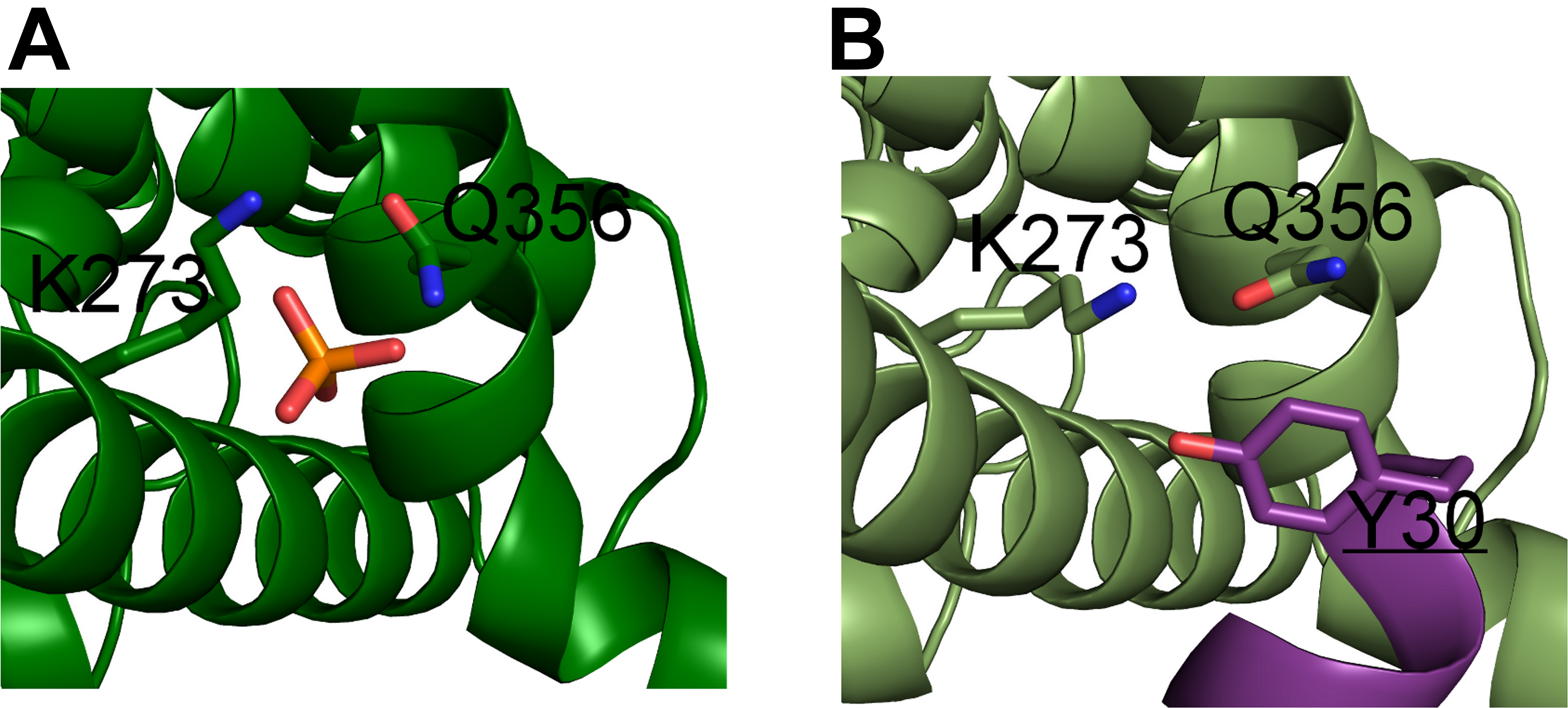

Supplement: Figure S3 — The sulfotyrosine binding site. DBP-RII molecules are in green and yellow. The bound DARC molecule is shown in purple. (A) Phosphate or selenate in the apo DBP-RII structure occupy the same position as (B) DARC Y30, defining the sulfotyrosine binding pocket. (TIFF) [file ppat.1003869.s003.tiff]

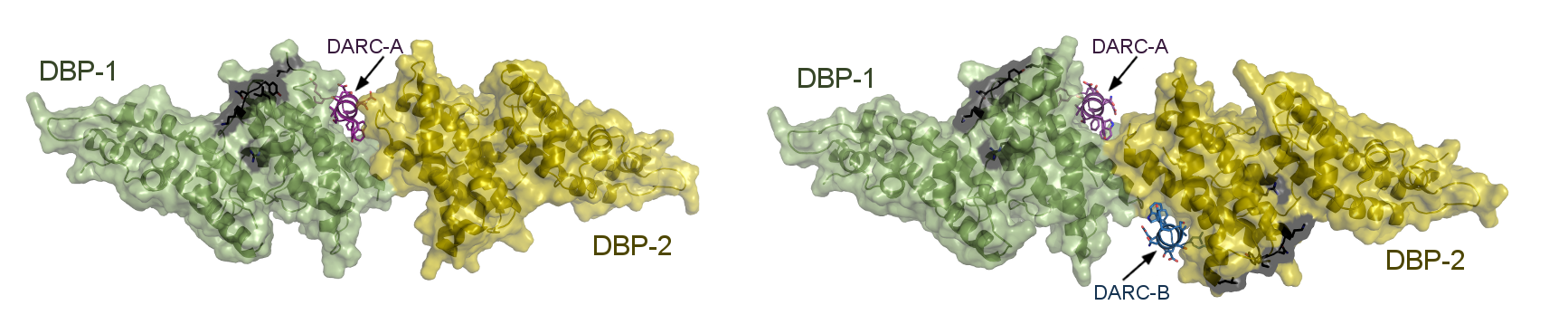

Supplement: Figure S4 — The DARC binding pockets are distinct from residues previously suggested to bind DARC from mutagenesis studies. DBP-RII monomers are in yellow and green. DARC monomers are in purple and blue. Residues previously suggested [40] to contact DARC are in black. (TIFF) [file ppat.1003869.s004.tiff]
